# Multi-Residue Method for Pesticides Determination in Dried Hops by Liquid Chromatography Tandem Mass Spectrometry

**DOI:** 10.3390/molecules28134989

**Published:** 2023-06-25

**Authors:** Marcin Gruba, Emilia Jóźwik, Mariusz Chmiel, Katarzyna Tyśkiewicz, Marcin Konkol, Anna Watros, Krystyna Skalicka-Woźniak, Grzegorz Woźniakowski

**Affiliations:** 1 Łukasiewicz Research Network—New Chemical Syntheses Institute, Al. Tysiąclecia Państwa Polskiego 13a, 24-110 Puławy, Poland; emilia.jozwik@ins.lukasiewicz.gov.pl (E.J.); mariusz.chmiel@ins.lukasiewicz.gov.pl (M.C.); katarzyna.tyskiewicz@ins.lukasiewicz.gov.pl (K.T.); marcin.konkol@ins.lukasiewicz.gov.pl (M.K.); anna.watros@ins.lukasiewicz.gov.pl (A.W.); 2 Department of Natural Products Chemistry, Medical University of Lublin, ul. Chodźki 1, 20-093 Lublin, Poland; krystyna.skalicka-wozniak@umlub.pl; 3 Department of Infectious and Invasive Diseases and Veterinary Administration, Institute of Veterinary Medicine, Faculty of Biological and Veterinary Sciences, Nicolaus Copernicus University in Toruń, Lwowska 1 Street, 87-100 Toruń, Poland; grzegorz.wozniakowski@umk.pl

**Keywords:** dried hops, hop cones, pesticide residues, QuEChERS, LC-MS/MS

## Abstract

In this study a multi-residue determination method for 36 pesticides in dried hops was reported. The sample preparation procedure was based on the acetate buffered QuEChERS method. A few mixtures of dispersive solid phase extraction (dSPE) sorbents consisting PSA, C_18_, GCB, Z-Sep and Z-Sep+ were investigated to clean-up the supernatant and minimize matrix co-extractives. The degree of clean-up was assessed by gravimetric measurements, which showed the best results for mixtures containing the Z-Sep+ sorbent. This is the first study to apply Z-Sep+ sorbent for hops material and the first to improve the method for pesticide residues determination in hops. Samples were analysed using liquid chromatography coupled with tandem mass spectrometry (LC-MS/MS) and the procedure was validated according to the SANTE/11813/2017 document at four concentration levels: 0.02, 0.05, 0.1 and 1 mg/kg. The limits of quantification (LOQ) were in the range of 0.02–0.1 mg/kg. For all active substances, the trueness (recovery) ranged from 70 to 120% and the precision (RSD_r_) value was <20%. Specificity, linearity and matrix effect were also evaluated. The validated method was applied to the analysis of 15 real dried hop samples and the relevant data on detected residues were included.

## 1. Introduction

Hop (*Humulus lupulus* L.) is a perennial climbing plant belonging to the same family as hemp (*Cannabaceae* L.) and the same order as nettles (*Urticales* L.). It is a dioecious plant producing both male and female inflorescences, characterized by twining vines that can reach from 3 m to 6 m in height. The leaves are arranged in opposite, with characteristic hairiness and spiky serration. Hop is widespread both in Europe (notably in Poland) and Central Asia. It is cultivated in many countries as an industrial plant used for brewing purposes. The habitat for wild hop includes moist thickets and river banks, as well as loamy, moist soils [1]. In the recent years, the hop production in the European Union has amounted to over 50,000 tons per year with Poland as the third largest producer of hop in Europe after Germany and Czechia [2].

**Figure 1:**
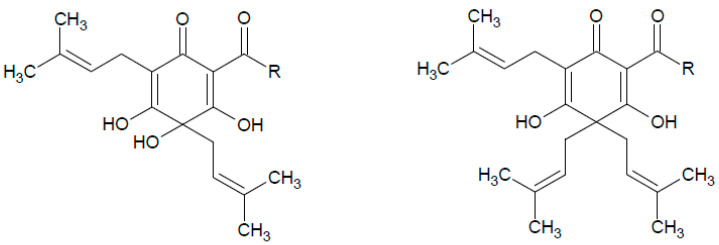
The chemical structure of alpha- and beta-acids.

Hop cones (hops) are a source of valuable bioactive compounds with, e.g., various phenolic compounds exhibiting antibacterial, antiviral and anticancer properties [3,4,5]. Due to the presence of alpha acids (Figure 1) in cones, which are responsible for bitterness [6], hops are mainly used in beer production. Moreover, the use of hop cones in the medicine and cosmetics industry has been also reported [7].

Hops, like many other crops, are exposed to various diseases, losses caused by pests and weed infestation. For this reason, a number of pesticides are used to protect the hop plant. However, the use of agricultural treatments generates problems connected with consumer health due to the persistence of pesticide residues in hops for a long time. Therefore, it is important to control the level of pesticide residues to ensure the safety of consumers. As for that, the maximum residue levels (MRLs) of pesticides have been established and regularly updated by European Commission [8].

The major components of hops are resins, essential oils, chlorophyll and phenolic compounds. Therefore, it is a complex matrix, for which an appropriate sample preparation and clean-up is required. In recent years, the QuEChERS method has been the most popular for multi-residue pesticides’ determination in food. This technique is based on acetonitrile extraction followed by a clean-up protocol with dispersive solid phase extraction (dSPE) on different sorbents, e.g., octadecylsilane (C_18_), primary secondary amine (PSA), graphitized carbon black (GCB), and recently zirconia-based sorbents (Z-Sep, Z-Sep+). The QuEChERS method was previously adopted by Dusek et al. [9,10] for the determination of pesticide residues in hops by liquid chromatography coupled with tandem mass spectrometry.

The aim of the present study was to develop and validate an improved multi-residue method for the determination of 36 pesticide residues in hop samples using LC-MS/MS. For the clean-up protocol and to minimize matrix co-extractives a few mixtures of sorbents, including Z-Sep+ (for the first time for hops matrix), were investigated. This method was used for the determination of pesticide residues in 15 sample batches of hops as a part of raw material quality control.

## 2. Results and Discussion

### 2.1. Optimization of Sample Preparation

Three versions of a QuEChERS extraction step, such as original unbuffered, citrate and acetate buffered are commonly used in labs for a wide range of pesticide residues in various matrices with good results for most pesticides [11]. However, the buffered methods are suggested by authors of original QuEChERS as a good extraction recovery of pH dependent analytes. An original unbuffered QuECHERS method [12] was published by Dusek et al. [9] for the determination of 48 pesticide residues in hops. The citrate buffered QuEChERS preparation protocol [13] for 56 pesticide residues in hops was published by the same authors [10]. In the proposed methodology in this paper, acetate buffered QuEChERS [14] was used for hop samples extraction.

The degree of dSPE clean-up was assessed by gravimetric measurements and then the content of co-extractives (corresponding to 1 mL in the final extract) was calculated.

In the first step, 3 mL of initial acetonitrile extract (in two repetitions) was transferred to pre-weighted glass vials. The moisture was removed by heating the vials for 1 h at 105 °C prior to weighing. After that, the extract was taken to dryness in nitrogen stream. Then, vials were heated again for 1 h at 105 °C to remove moisture and weighed. The same procedure was applied to final extracts cleaned by various sorbent mixtures. The difference in weight was assessed as the amount of co-extractives in the initial and final extracts. Table 1 presents the various mixtures used to optimize the dSPE clean-up step and obtained results.

The Z-Sep sorbent is zirconium dioxide bonded to silica, whereas Z-Sep+ is a zirconium dioxide and C_18_ dual bonded to silica. These sorbents were proposed as an alternative for PSA to lipids removal in high-content oil samples by Rajski et al. [15]. In this study, Z-Sep was shown to remove more co-extracted matrix components than PSA and C_18_. Other authors reported that PSA and C_18_ in fish tissue sample removed most co-extractives by weight, however Z-Sep provided better trueness and precision [16]. The combination of PSA and Z-Sep+ was tested for dSPE clean-up in the determination of pesticide residues in honeybees [17]. The authors showed that clean-up mechanism of PSA is especially based on pH dependent ionic interactions, whereas Z-Sep+ is based on pH independent Lewis acid and hydrophobic interactions. The combination of these two sorbents in dSPE clean-up protocol of honeybee samples, consisting mainly of waxes and proteins, provided an excellent result. The combination of PSA, C_18_ and Z-Sep was selected by Dusek et al. [9] for the sample preparation procedure of dried hops, but in the mentioned work the Z-Sep+ sorbent was not tested at all.

In the present study, several mixtures of sorbents were tested for the efficiency of removing co-extracted matrix components. The PSA and Z-Sep+ mixture (C) in the same amount as PSA and C_18_ (A) showed almost 42% less matrix co-extractives. As compared to PSA and Z-Sep (B), the difference was also significant and equalled 34.5%.

The GCB sorbent is very effective for QuEChERS extracts clean-up, but retains pesticides with planar structures [14,18]. Due to the high content of chlorophyll in the hop samples, a small amount of GCB was used for an effective removal of this group of compounds. On the other hand, the procedural standard calibration was applied to compensate low extraction recoveries of planar pesticides such as thiabendazole. It should be mentioned that the extract after dSPE clean-up with GCB was better purified than in the absence of GCB sorbent, and the amount of co-extractives in this case was the lowest.

Finally, a mixture (D) of dSPE sorbents containing 50 mg MgSO_4_, 50 mg PSA, 50 mg Z-Sep+ and 5 mg GCB, which showed 47% less co-extractives than mixture (A), was chosen for the validation and routine analysis.

### 2.2. Validation of Analytical Procedure

The validation data, which are presented in Table 2, met the criteria of the SANTE/11813/2017 guidance document.

The LOQ values were in the range of 0.02–0.05 mg/kg for 89% of pesticides. Only for boscalid, hexythiazox, quinoxyfen and spirodiclofen the LOQs were equal to 0.1 mg/kg, but for all of the analytes these values were lower or equal to the MRLs.

A number of substances present in hops were co-extracted with the analytes during the sample preparation. Due to the co-extraction of matrix components in LC-MS/MS, the analyte signal might be enhanced and more often suppressed [19]. This phenomenon known as the matrix effect could affect the quality and performance of the LC-MS/MS analysis [20]. In the present study, the matrix effect was examined without an internal standard because there are reports that it may affect the results [11]. The signal enhancement was not observed for any analyte; however, suppression was demonstrated for all of the analytes. Negligible matrix effect (from −20% to 20%) was showed by 14% of compounds. Medium matrix effect (from 21% to 50% positive values and from −21% to −50% negative values) was proved for 53% of compounds, and a strong matrix effect (above 50% positive values and below −50% negative values) was observed for 33% of analytes. To overcome this phenomenon the procedural standard calibration was used.

The average precision represented as relative standard deviation (RSD_r_) and trueness (recovery) were calculated at the levels of 0.02, 0.05, 0.1 and 1 mg/kg based on five replications for each level. For all of the analytes RSD_r_ values were <20% and recovery ranged from 70 to 120%.

Calibration curves were prepared based on the procedural standard calibration. For this purpose, for each level, the blank samples before extraction were spiked in two repetitions with an appropriate amount of standard solution and internal standard for ensuring accuracy of the method. The data showed good linearity for all of the pesticides in the range of LOQ-4 mg/kg with a correlation coefficient (R^2^) range 0.989–0.999.

**Table 2:** List of analytes according to categories [21]: A—acaricide, F—fungicide, H—herbicide, I—insecticide, MRL for hops [8] and results of validation parameters (LOQ, matrix effect without internal standard, linearity, trueness and precision).

Analyte	Category	MRL (mg/kg)	LOQ (mg/kg)	ME (%) w/o IS	Linearity (mg/kg)	R^2^	Recovery, % (RSD_r_, %)			
							0.02 mg/kg	0.05 mg/kg	0.1 mg/kg	1 mg/kg
Acetamiprid	I	0.05	0.02	−53	0.02–4	0.999	100 (8)	98 (5)	101 (9)	92 (3)
Azoxystrobin	F	30	0.02	−22	0.02–4	0.998	84 (3)	90 (6)	91 (4)	90 (3)
Bifenazate	I	20	0.05	−60	0.05–4	0.996		108 (10)	113 (5)	104 (9)
Boscalid	F	80	0.1	−56	0.1–4	0.995			108 (7)	98 (10)
Carbofuran	I	0.05	0.02	−28	0.02–4	0.998	110 (8)	105 (4)	105 (2)	94 (2)
Chloridazon	H	0.1	0.05	−39	0.05–4	0.999		114 (2)	106 (5)	90 (4)
Chlorotoluron	H	0.05	0.02	−33	0.02–4	0.999	112 (4)	107 (1)	109 (1)	101 (1)
Diazinon	I	0.5	0.05	−58	0.05–4	0.996		98 (7)	99 (3)	89 (6)
Difenoconazole	F	0.05	0.05	−40	0.05–4	0.999		111 (7)	110 (5)	96 (4)
Dimethoate	I	0.05	0.02	−25	0.02–4	0.999	119 (4)	108 (2)	105 (3)	89 (4)
Dimethomorph	F	80	0.05	−9	0.05–4	0.998		116 (5)	106 (6)	89 (4)
Epoxiconazole	F	0.1	0.05	−33	0.05–4	0.995		112 (7)	110 (6)	92 (8)
Etoxazole	I	15	0.05	−54	0.05–4	0.999		85 (5)	92 (3)	84 (2)
Fenamidone	F	0.05	0.05	−34	0.05–4	0.994		102 (8)	97 (7)	85 (9)
Fenhexamid	F	0.05	0.05	−45	0.05–4	0.997		100 (7)	96 (8)	81 (7)
Fenpyroximate	A	15	0.05	−65	0.05–4	0.998		107 (7)	99 (4)	85 (3)
Flufenoxuron	I	0.05	0.05	−70	0.05–4	0.991		111 (5)	108 (7)	92 (1)
Flusilazole	F	0.05	0.05	−48	0.05–4	0.994		96 (13)	100 (7)	92 (6)
Hexythiazox	A	20	0.1	−86	0.1–4	0.989			104 (3)	102 (5)
Imidacloprid	I	10	0.02	−18	0.02–4	0.999	114 (6)	108 (6)	101 (8)	93 (5)
Isoproturon	H	0.05	0.02	−14	0.02–4	0.999	95 (1)	101 (4)	103 (4)	96 (1)
Linuron	H	0.05	0.05	−50	0.05–4	0.997		117 (4)	111 (5)	102 (3)
Mandipropamid	F	90	0.05	−26	0.05–4	0.998		110 (4)	107 (4)	91 (3)
Metalaxyl	F	15	0.02	−43	0.02–4	0.998	89 (10)	94 (9)	95 (4)	89 (4)
Myclobutanil	F	6	0.05	−45	0.05–4	0.998		106 (10)	95 (5)	80 (7)
Picoxystrobin	F	0.05	0.02	−24	0.02–4	0.995	99 (9)	107 (7)	110 (5)	101 (4)
Propargite	A	0.05	0.05	−69	0.05–4	0.997		92 (12)	97 (7)	82 (5)
Pyraclostrobin	F	15	0.05	−58	0.05–4	0.998		105 (6)	111 (7)	99 (4)
Pyrimethanil	F	0.05	0.05	−18	0.05–4	0.995		114 (3)	109 (4)	93 (5)
Quinoxyfen	F	2	0.1	−75	0.1–4	0.994			103 (7)	83 (5)
Spirodiclofen	A	40	0.1	−58	0.1–4	0.997			116 (9)	99 (4)
Spirotetramat	I	15	0.05	−2	0.05–4	0.998		104 (8)	108 (7)	98 (6)
Tebuconazole	F	40	0.05	−50	0.05–4	0.996		79 (12)	89 (5)	92 (7)
Thiabendazole	F	0.05	0.02	−27	0.02–4	0.993	97 (11)	106 (3)	107 (8)	100 (4)
Thiacloprid	I	0.05	0.02	−21	0.02–4	0.999	105 (2)	107 (3)	104 (3)	95 (3)
Trifloxystrobin	F	40	0.02	−47	0.02–4	0.999	113 (6)	106 (2)	103 (3)	97 (4)

### 2.3. Real Samples

**Figure 2:**
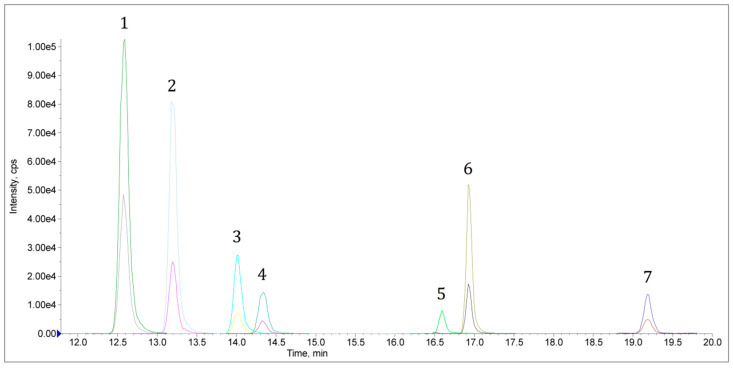
LC-MS/MS (sMRM) chromatogram of a real, dried hop sample with internal standard and six pesticide residues detected. 1—atrazine D_5_ (IS), 2—azoxystrobin, 3—mandipropamid, 4—boscalid, 5—pyraclostrobin, 6—trifloxystrobin, 7—spirodiclofen.

All of the fifteen analysed dried hop samples contained pesticide residues. One or two pesticide residues were detected in six samples, three residues were found in seven samples and five residues in one sample. A maximum of six residues was also found in one sample (Figure 2). However, in accordance with the European Commission limits [8], the MRLs were not exceeded in any case. In total, residues of twelve pesticides were detected in all samples. The highest detected residue level in one of the samples was for boscalid (9.4 mg/kg) but in this case the MRL for dried hops is equal to 80 mg/kg. In addition, in other samples, high concentrations of mandipropamid (7.3 mg/kg), dimethomorph (3.4 mg/kg), azoxystrobin (2.2 mg/kg) and pyraclostrobin (2.1 mg/kg) were detected, but the MRL limits for residues of these pesticides are 90, 80, 30 and 15 mg/kg, respectively. In the tested samples, residues of fungicides such as azoxystrobin, boscalid, pyraclostrobin and trifloxystrobin were the most dominant. Similar results were obtained in a study performed in Czechia, where 24 field samples of cone hops were analyzed [10]. The residues of fungicides such as mandipropamid, boscalid, azoxystrobin, pyraclostrobin, dimethomorph and ametoctradin were determined and the highest individual residue concentration was 31 mg/kg for mandipropamid [10]. The reason for the frequent detection of fungicide residues may be related to the fact that hop diseases caused by different types of fungi are among the most common in Europe [22]. Detailed data on the detected residues in analysed samples are presented in Table 3.

## 3. Materials and Methods

### 3.1. Standards and Reagents

Pesticide analytical standards (acetamiprid, azoxystrobin, bifenazate, boscalid, carbofuran, chloridazon, chlorotoluron, diazinon, difenoconazole, dimethoate, dimethomorph, epoxiconazole, etoxazole, fenamidone, fenhexamid, fenpyroximate, flufenoxuron, flusilazole, hexythiazox, imidacloprid, isoproturon, linuron, mandipropamid, metalaxyl, myclobutanil, picoxystrobin, propargite, pyraclostrobin, pyrimethanil, quinoxyfen, spirodiclofen, spirotetramat, tebuconazole, thiabendazole, thiacloprid, trifloxystrobin) and internal standard (atrazine D_5_), all 97% or higher purity, were purchased from Sigma-Aldrich (Seelze, Germany) and the Institute of Industrial Organic Chemistry (Warsaw, Poland). Stock standard solutions (1 mg/mL) were prepared in acetone and stored at −18 °C. Individual and mixed standard solutions for optimization and experiments were prepared from the stock standards. Acetonitrile (LC-MS grade) used for a QuEChERS extraction step and acetone (HPLC grade) used for the standards preparation were obtained from VWR Chemicals (Radnor, PA, USA). Methanol (LC-MS grade) used as eluent in liquid chromatography, glacial acetic acid used as a mobile phase additive and one sample preparation solution were purchased from Avantor Performance Materials Poland S.A. (Gliwice, Poland). Ultra-pure water from Labconco Water Pro Ps (Kansas City, MO, USA) system was used for mobile phase and for the sample preparation. Ammonium formate used as a mobile phase addition, anhydrous magnesium sulfate (MgSO_4_) and anhydrous sodium acetate (C_2_H_3_NaO_2_) used for the QuEChERS extraction step were obtained from Sigma-Aldrich (Steinheim, Germany). For the clean-up step C_18_, PSA, Z-Sep, Z-Sep+ sorbents from Sigma-Aldrich (Bellefonte, PA, USA) and GCB sorbent from Phenomenex (Torrance, CA, USA) were purchased.

### 3.2. Instrumentation

For LC analysis, an Agilent 1200 Series (Waldbronn, Germany) liquid chromatograph equipped with a reversed phase C_18_ (Agilent Zorbax Eclipse XDB 4.6 mm × 150 mm, 5 µm particle size) column was applied. The column oven temperature was 40 °C and the analytes were separated using water (mobile phase A) and methanol (mobile phase B), both with 5 mM ammonium formate and 0.1% acetic acid. The flow rate was 0.5 mL/min and gradient program was made as follows: 20% B increased to 64% in 1 min, increased to 80% in 8 min, increased to 95% in 2 min and held for 10 min, decreased to 20% in 1 min and held for 10 min for column recondition. The total analysis run time was 32 min, and the injection volume was 10 µL. For MS/MS analysis, an AB Sciex 3200 QTRAP (Framingham, MA, USA) triple quadrupole mass spectrometer equipped with an electrospray ionisation source (ESI) was used. The ion source worked in positive ionisation mode and the scheduled multiple reaction monitoring (sMRM) was applied. The ion source settings were as follows: temperature—600 °C, ion spray voltage—5500 V, curtain gas—20 psi, collision gas—medium, ion source gas 1–45 psi, ion source gas 2–55 psi. The lC-MS/MS system was controlled by Analyst Software (version 1.5.2).

### 3.3. Sample Preparation

Homogenized dried hops (0.5 g) were weighed in a 50 mL centrifuge tube followed by the addition of 50 μL of the internal standard (10 μg/mL—atrazine D_5_). Then, the sample was mixed with 5 mL of water using a vortex device for 30 s. In the next step, 10 mL of 1% acetic acid in acetonitrile was added and shaken manually for 1 min. After that, the sample was cooled in the freezer for 30 min. In the next step, a salting mixture (4 g of anhydrous MgSO_4_ and 1 g of anhydrous C_2_H_3_NaO_2_) was added, shaken manually for 1 min and centrifuged for 5 min at 4000 rpm. Then, 500 µL of supernatant was removed and cleaned by dispersive solid phase extraction mixture. The composition of the dSPE mixture, which was finally chosen, is as follows: 50 mg of anhydrous MgSO_4_, 50 mg of PSA, 50 mg of Z-Sep+ and 5 mg of GCB. The supernatant was shaken manually for 2 min and centrifuged again. Before injection into LC-MS/MS system the extract was filtered through a 0.2 μm syringe PTFE filter.

### 3.4. Validation of the Analytical Procedure

The initial full validation was conducted in accordance with the SANTE/11813/2017 guidelines, included on page 26 of this document [23]. Specificity, linearity, matrix effect, limit of quantification, trueness and precision were evaluated. The dried hop samples free from pesticide residues were used to prepare procedural standard calibration and used as blank in order to spike aliquots for validation studies. The limit of quantification (LOQ) was set as the minimum concentration that can be quantified with acceptable precision and trueness by spiking sample at this concentration level. Linearity was evaluated in duplicate at six concentration levels: 0.02, 0.05, 0.1, 1, 2 and 4 mg/kg. Spiked dried hop samples in five replicates for each level were used to estimate precision and trueness. The matrix effect (ME%) was examined by comparing the slopes of the calibration curves in pure solvent and matrix-matched without the internal standard using the following equation:

(1) $$ME\%=\left(\frac{slope\;of\;matrix\;matched\;calibration\;curve}{slope\;of\;calibration\;curve\;in\;pure\;solvent}-1\right)\times 100$$

### 3.5. Real Samples

The developed and validated method was applied to analyse pesticide residues in 15 sample batches of Hallertauer Magnum variety dried hop, harvest year 2019, from Powiśle Lubelskie Region (southeast Poland). Subsequently, the supercritical CO_2_ hop extracts were made from hops after pelleting.

## 4. Conclusions

Hop cones are the source of valuable bitter acids, essential oils and polyphenols [24]. On the other hand, they are also a complex matrix. As suggested by Hrnčič et al. [25], even the isolation of chemical compounds and their analyses are time-consuming and challenging due to the complexity of the hop cones matrix. Given the advantage of pesticide residue methods used so far, none of them tested Z-Sep+ for hop cones in order to remove matrix co-extractives.

The improved and validated method for determination of pesticide residues was successfully applied for dried hop samples by liquid chromatography coupled with tandem mass spectrometry. The combination of QuEChERS extraction and dSPE clean-up protocol with a mixture of PSA and Z-Sep+ enabled a decrease in co-extracted matrix components to a higher extent than with PSA and C_18,_ and PSA and Z-Sep. In addition, the use of a small amount of GCB effectively removed chlorophyll from the hop matrix, and the validation results showed satisfactory values for the precision and trueness.

The overall idea of pesticide residue testing is to assure end-users about the quality of product ingredients. According to the obtained results, all pesticides’ residues (most belonging to fungicides) were detected at a level much lower than the allowable quantities. The proposed analytical method ensures consumer safety and brewing quality in further application steps and can be useful as an element of routine quality control of raw materials in the production process of supercritical CO_2_ hop extract for brewing applications.

## Figures and Tables

**Table 1:** Content of matrix co-extractives in QuEChERS extracts before (no clean-up) and after dSPE clean-up protocol (mixtures A–D).

Clean-Up Mixtures	Content of Matrix Co-Extractives (mg/mL)
No clean-up	12.32 ± 0.07
(A) 50 mg MgSO_4_, 50 mg PSA, 50 mg C_18_	4.50 ± 0.09
(B) 50 mg MgSO_4_, 50 mg PSA, 50 mg Z-Sep	4.00 ± 0.14
(C) 50 mg MgSO_4_, 50 mg PSA, 50 mg Z-Sep+	2.62 ± 0.12
(D) 50 mg MgSO_4_, 50 mg PSA, 50 mg Z-Sep+, 5 mg GCB	2.37 ± 0.05

**Table 3:** Pesticide residues in 15 samples of Hallertauer Magnum dried hop arranged according to frequency of occurrence.

Analyte	Number of Samples Containing	Range of Detected Residues (mg/kg)	MRL (mg/kg)
Azoxystrobin	9	0.030–2.2	30
Boscalid	7	0.16–9.4	80
Pyraclostrobin	6	0.065–2.1	15
Trifloxystrobin	6	0.020–0.76	40
Spirodiclofen	3	0.29–1.2	40
Metalaxyl	3	0.040–0.052	15
Mandipropamid	2	0.84–7.3	90
Dimethomorph	1	3.4	80
Myclobutanil	1	0.98	6
Quinoxyfen	1	0.67	2
Imidacloprid	1	0.093	10
Fenpyroximate	1	0.064	30

MRL—maximum residue level.

## Data Availability

The data presented in this study are available in this manuscript.
